# Inhibition and Reversal of Microbial Attachment by an Antibody with Parasteric Activity against the FimH Adhesin of Uropathogenic *E*. *coli*

**DOI:** 10.1371/journal.ppat.1004857

**Published:** 2015-05-14

**Authors:** Dagmara I. Kisiela, Hovhannes Avagyan, Della Friend, Aachal Jalan, Shivani Gupta, Gianluca Interlandi, Yan Liu, Veronika Tchesnokova, Victoria B. Rodriguez, John P. Sumida, Roland K. Strong, Xue-Ru Wu, Wendy E. Thomas, Evgeni V. Sokurenko

**Affiliations:** 1 Department of Microbiology, University of Washington, Seattle, Washington, United States of America; 2 Division of Basic Sciences, Fred Hutchinson Cancer Research Center, Seattle, Washington, United States of America; 3 Department of Bioengineering, University of Washington, Seattle, Washington, United States of America; 4 Department of Urology, New York University School of Medicine, New York, New York, United States of America; 5 Analytical Biopharmacy Core, University of Washington, Seattle, Washington, United States of America; University of California, Davis, UNITED STATES

## Abstract

Attachment proteins from the surface of eukaryotic cells, bacteria and viruses are critical receptors in cell adhesion or signaling and are primary targets for the development of vaccines and therapeutic antibodies. It is proposed that the ligand-binding pocket in receptor proteins can shift between inactive and active conformations with weak and strong ligand-binding capability, respectively. Here, using monoclonal antibodies against a vaccine target protein - fimbrial adhesin FimH of uropathogenic *Escherichia coli*, we demonstrate that unusually strong receptor inhibition can be achieved by antibody that binds within the binding pocket and displaces the ligand in a non-competitive way. The non-competitive antibody binds to a loop that interacts with the ligand in the active conformation of the pocket but is shifted away from ligand in the inactive conformation. We refer to this as a parasteric inhibition, where the inhibitor binds adjacent to the ligand in the binding pocket. We showed that the receptor-blocking mechanism of parasteric antibody differs from that of orthosteric inhibition, where the inhibitor replaces the ligand or allosteric inhibition where the inhibitor binds at a site distant from the ligand, and is very potent in blocking bacterial adhesion, dissolving surface-adherent biofilms and protecting mice from urinary bladder infection.

## Author Summary

A common approach in the development of selective inhibitors for ligand-receptor interactions is targeting the receptor binding site with the expectation that inhibitors will sterically interfere with ligand binding and thus block receptor function via a competitive (orthosteric) mechanism. However, using monoclonal antibodies specific for the mannose-binding *Escherichia coli* adhesin, FimH, we demonstrate that the binding site epitopes allow for non-competitive inhibition that is more effective than orthosteric blocking. FimH, similar to other binding proteins, exhibits conformational flexibility of the ligand-binding pocket shifting between open (inactive) and tight (active) conformations, with relatively low- and high- affinity towards mannose. We show that an antibody that binds just one of the mannose-binding pocket loops prevents the shift from the inactive to the active conformation and hence blocks formation of high-affinity ligand-receptor complexes. This antibody type was more effective in inhibition of bacterial adhesion than anti-FimH antibodies competitively blocking mannose binding, and unlike the latter or a soluble ligand, showed the ability to detach an established bacterial biofilm from a ligand-coated surface. As the newly described antibody can bind the FimH pocket simultaneously with ligand, we refer to it as a parasteric (next-to-ligand) inhibitor that exhibits non-competitive inhibition from within the binding-pocket of the receptor.

## Introduction

Receptor-ligand interactions are among the most basic biological phenomena involved in cell signaling, adhesion and pathogen attachment. Antibody- or small molecule-based inhibitors of these interactions are of great importance for various preventive and therapeutic implications, including development of protective vaccines. Two general types of inhibitory mechanisms have been described to date. Orthosteric inhibitors directly compete with ligands for the binding pocket and, thus, their receptor-inhibitory activity is of a competitive nature [1]. In contrast, allosteric inhibitors exert their effects via interaction with a site that is separate from the ligand-binding pocket and accomplish the inhibition in a non-competitive manner [2]. Non-competitive inhibition is less sensitive to endogenous ligand and thus is generally more effective pharmacologically [3]. In the current study, we describe a type of inhibitory monoclonal antibody against the mannose-binding adhesin of *E*. *coli*, FimH, that does not fall into either of the expected two categories of inhibitors. Like an allosteric inhibitor, this antibody exerts non-competitive inhibition, but like an orthosteric inhibitor, it binds within the ligand-binding pocket. Unlike the latter, however, it forces the conversion of the binding pocket to an open, inactive conformation, even when the pocket is occupied by the ligand mannose.

FimH is a 30 kDa lectin-like protein that is incorporated into the tip of surface hair-like structures of *E*. *coli* and other enterobacteria called type 1 fimbriae [4]. It exhibits specificity to glycoproteins carrying terminally exposed mannose and is critical for the virulence of uropathogenic strains of *E*. *coli* [5,6,7,8,9]. FimH has two domains: the C-terminal pilin domain that anchors the adhesin to the fimbrial rod and the N-terminal lectin domain that is responsible for mannose binding [10]. The binding pocket in the lectin domain shifts between open and tightened conformations with low (K_D_ = 298 μM)- and high (K_D_ = 1.2 μM)- affinity for mannose, respectively [11,12,13]. The low-affinity (inactive) state of the lectin domain is allosterically stabilized by its interaction with the pilin domain that sustains a finger-trap-like twist in the β-sheets of the binding domain [11]. The high-affinity (active) state is induced by ligand binding and/or separation of the domains, with the latter facilitated by force during bacterial adhesion under flow conditions. FimH-like force-activated adhesion has been described in several other adhesive systems of different bacterial species as well as eukaryotic cells. For example, proteins like integrins [14] or P/L-selectins [15] exhibit a shift between inactive and active conformations under shear force.

The existence of two alternative conformations of the mannose-binding pocket of FimH reflects a broad phenomenon in the biology of receptor-ligand interactions, including enzyme binding to substrates and products. In fact, the century-old static ‘lock-and-key’ model of the interaction mechanism is considered now to be too rigid for many if not the majority of receptor proteins and enzymes. It has been shown that ligand-binding pockets are typically composed of residues on flexible loops and dynamically shift between active and inactive conformations, with relatively high and low (often unmeasurable) affinity for the ligand, respectively [16,17,18,19,20]. Generally, the ligand-bound active pocket assumes a more contracted shape than the ligand-free inactive pocket, so the corresponding receptor conformers are commonly referred to as open vs closed (or tightened) states [20,21,22,23]. Some well-studied examples of receptors with such pocket dynamics include allosteric proteins such as maltose-binding protein [24,25,26], and G-protein-coupled receptors (GPCRs) [21,23,27].

Two general models have been proposed to describe the effect of ligand on the conformation of receptor binding pockets. In the ‘induced fit’ model, the active state of the pocket is assumed only after ligand binds to the inactive state, while in the ‘conformational selection’ model, the inactive and active states coexist in the absence of ligand, but the active state is stabilized by ligand binding [28,29,30]. More complex models of ligand-receptor recognition that combine the two models are also considered [31]. All models allow for initial weak interaction of the ligand with the inactive state of binding pocket, and this weak interaction has been repeatedly shown to involve only a subset of the receptor residues that interact with the ligand in the strongly-binding active state [16,20,22,23,31].

Partial interaction of the ligand with the binding pocket leaves the remaining residues, which only interact with the ligand when the pocket is in the active state, free in theory to bind to an additional compound. Such a compound could potentially act as an inhibitor by interfering with the switch of the pocket into the active state. Such an inhibitor would not fit the accepted definition of either orthosteric inhibitors that cannot bind simultaneously with ligand, or allosteric inhibitors that should bind away from the binding pocket. Instead, because such inhibitors would bind next to the ligand, they could be classified as parasteric inhibitors. Interestingly, the parasteric binding of effector molecules and ligand was predicted previously for an enzymatic protein, low-molecular-weight acid phosphatase 1 (ACP1), though the structural basis or mechanism was not investigated [32].

In the current study we compared the inhibitory mechanism of different anti-FimH antibodies and describe an antibody that blocks the adhesive function in a distinct manner consistent with the parasteric model of inhibition. Compared to an orthosteric antibody, the parasteric antibody was a more potent inhibitor against bacterial adhesion, surface-bound biofilms and in vivo colonization, demonstrating that design of parasteric inhibitors potentially represents a very powerful approach toward the development of anti-adhesive preventive and therapeutic strategies.

## Results

### Anti-FimH antibodies mAb926 and mAb475 inhibit bacterial adhesion with different efficiencies and mechanisms

In a previous study, we characterized anti-FimH inhibitory antibodies raised against the lectin domain of FimH in the inactive conformation and described in detail the mAb475 antibody that directly competes with mannose binding [33]. Using IMGT/V-Quest software [34,35] we now have compared the germline origins of mAb475 and several other FimH-inhibiting monoclonal antibodies. One of the antibodies, mAb926, was of a different germline origin from mAb475, with the amino acid sequence homology of the mAb V-regions being only 55% and all three complementarity-determining regions being highly diverse (S1 Fig). We therefore compared the abilities of mAb926 and mAb475 to inhibit FimH in greater detail.

**Fig 1:**
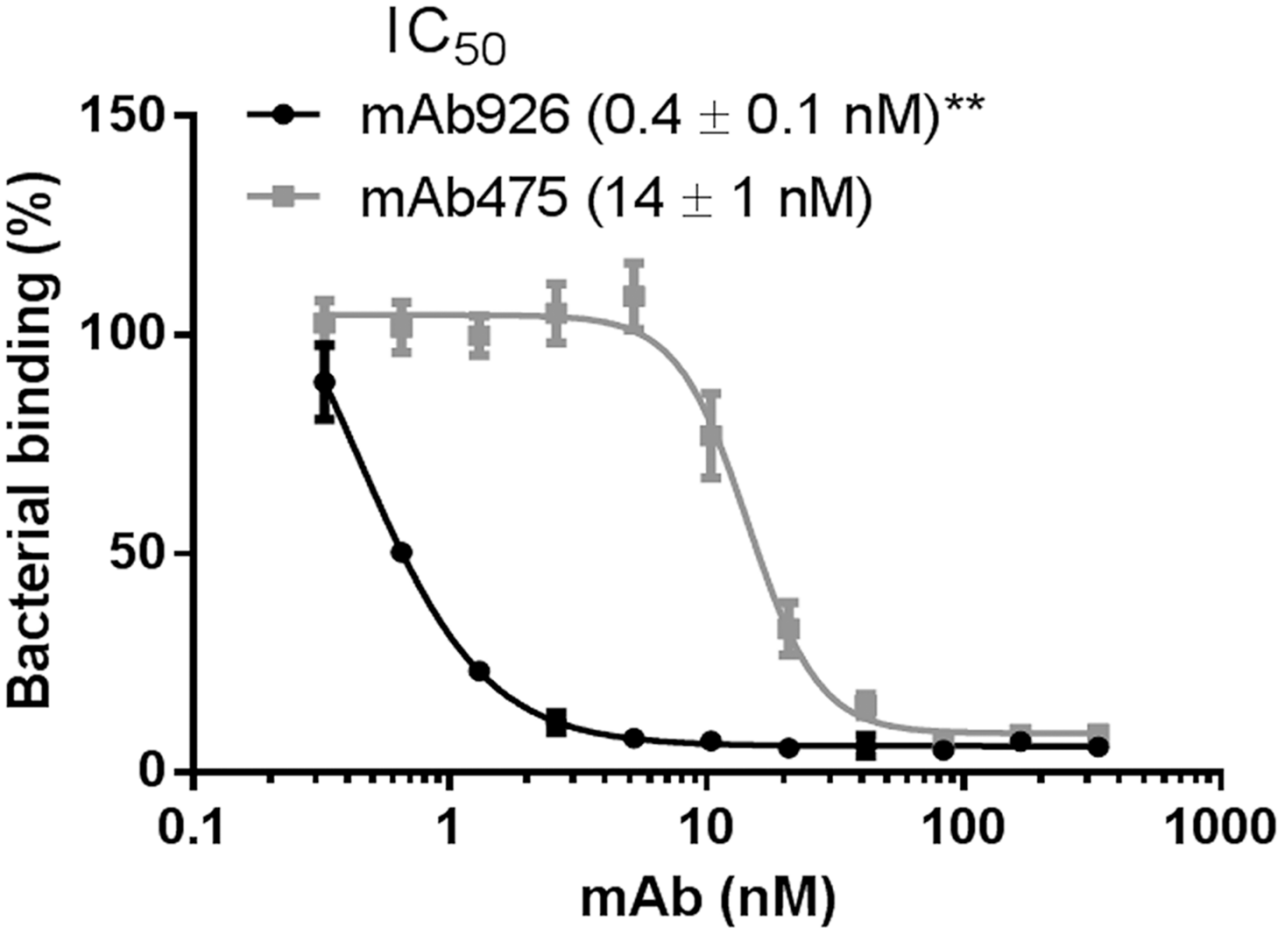
Inhibitory potency of mAb926 and mAb475. Binding of FimH^wt^-expressing *E*. *coli* to surface-immobilized yeast mannan in the presence of different concentrations of anti-FimH antibodies. Data are mean ± SEM (n = 5 independent experiments). The IC_50_ values were calculated from the fitted curves shown using Prism GraphPad 6 software. **, *P* ≤ 0.005 (t-test).

Type 1 fimbriated bacterial cells expressing the wild-type FimH adhesin variant of the uropathogenic *E*. *coli* strain J96 (FimH^wt^) were pre-incubated with different concentrations of the antibodies and then allowed to bind to mannosylated surfaces (Fig 1). mAb926 inhibited bacterial adhesion with half-maximal inhibitory concentration (IC_50_) of 0.4 ± 0.1 nM and mAb475 with IC_50_ of 14 ± 1 nM indicating much higher inhibitory potency of mAb926 (Fig 1). To determine if the 32-fold difference (*P*≤0.005) in inhibitory potency of the antibodies reflected a difference in binding affinity, we characterized binding of the antibodies to the purified fimbriae carrying FimH^wt^ with surface plasmon resonance. As shown in S2 Fig, the *K*_D_ of mAb926 was 7-fold lower than *K*_D_ of mAb475 (0.58 vs. 4.15 nM, respectively). Thus, the difference in IC_50_ between the two antibodies could not be explained by a difference in affinity. Indeed, mAb926 demonstrates >50% inhibition at its *K*_D_ concentration, while mAb475 demonstrates no measurable inhibition at its *K*_D_ concentration (Fig 1). Moreover, because mAb926 had a 17.6-fold higher association rate relative to mAb475 (49.7 vs. 2.8 x10^4^ M^-1^s^-1^, respectively), and a 2.5-fold higher dissociation rate (2.89 vs. 1.17 x10^-4^s^-1^, respectively), the difference in affinity was due to the faster association rate of mAb926. The SPR experiments were performed in parallel at the Analytical Biopharmacy Core, at the University of Washington (with separate preparations of the antigenic substrate), with results that are completely consistent with those reported here, adding confidence to our analyses (S1 Table). Since in the inhibition assay the antibodies were pre-incubated with the bacteria for one hour, the saturation of binding has likely been reached for both antibodies. Thus, at that point the difference in the dissociation rate of the inhibitory antibodies would be more important than their association rates, so the increased effectiveness of mAb926 is even more remarkable considering its slightly higher dissociation rate.

Thus, taken together, these results demonstrate the significantly higher inhibitory potential of mAb926 than of mAb475 antibodies cannot be explained by differences in binding kinetics or affinity of the two antibodies. Instead, the large difference in inhibitory potency must reflect some unknown difference in the mechanism of inhibition.

### Mannose directly competes with mAb475 but not mAb926 binding

**Fig 2:**
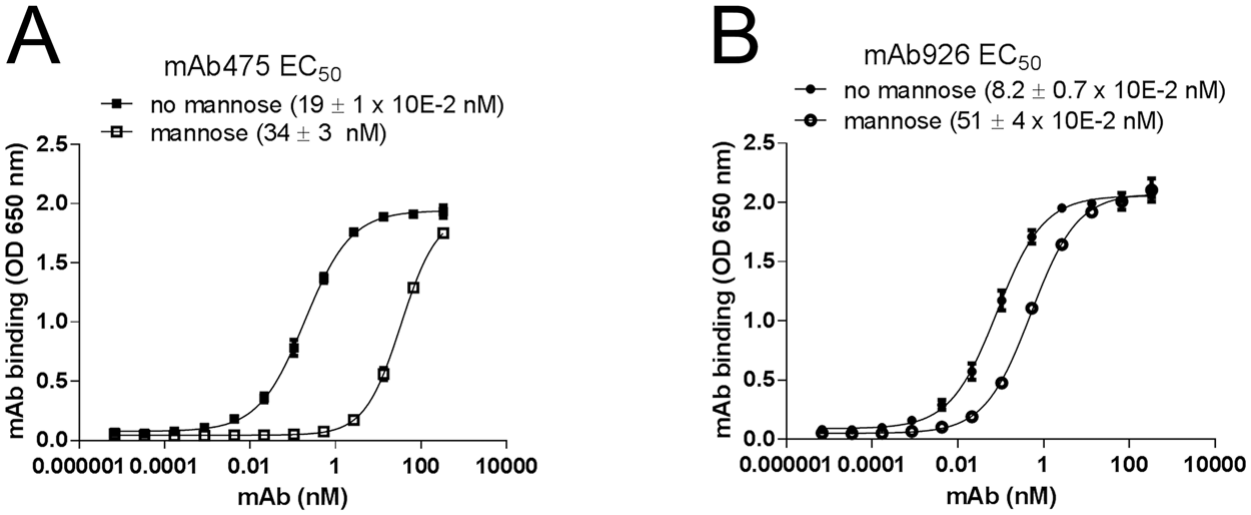
Effect of mannose on antibody binding. Dose-response curves of mAb475 (A) and mAb926 (B) binding to fimbrial FimH^wt^ in the absence and presence of 1% (52 mM) mannose. The EC_50_ values were calculated for each mAb separately from the fitted curves shown using Prism Graphpad 6 software. Data shown are mean ± SEM (n = 3 independent experiments).

We next compared the ability of mAb475 and mAb926 to bind FimH in the presence of soluble mannose. As shown in Fig 2A, mannose strongly inhibited mAb475 binding, causing a significant shift of its binding curve towards higher concentrations of the antibody. The mAb475 half-maximal effective concentration (EC_50_) increased 179-fold in the presence of soluble mannose. In contrast, binding of mAb926 was affected by mannose to a much lesser extent resulting in a relatively small rightward shift of the binding curve with a 6.2-fold increase in the mAb926 EC_50_ (Fig 2B).

To distinguish between competitive versus non-competitive interactions of mannose and the antibodies, we compared the observed EC_50_ ratio values for antibody binding with an EC_50_ ratio for a model of two ligands binding to a receptor [36]. Based on the model, mannose and antibody can compete for binding to the same site on FimH according to their relative concentrations and affinities, or bind to the receptor simultaneously (S3 Fig) with the affinities altered by a cooperativity factor α [37]. The calculated EC_50_ ratio for competitive binding (see Material and Methods) was 175 ± 30, which is consistent with the EC_50_ ratio experimentally determined for mAb475 (179) confirming that the antibody is a direct competitor. Thus, binding of mAb475 and mannose to FimH is not simultaneous but mutually exclusive, implying binding to a structurally identical site, consistent with our previously reported determination of the mAb475 epitope [33]. However, the 6.2-fold alteration of mAb926 EC_50_ by mannose cannot be explained by the competitive inhibition model but instead indicates that mannose inhibits mAb926 binding in a non-competitive manner consistent with simultaneous binding of the antibodies and mannose to FimH.

The non-competitive relationship of mAb926 with the mannose ligand indicates that, unlike with mAb475, inhibition of FimH activity by mAb926 is not via a direct orthosteric mechanism. It rather resembles more the mechanism exerted by allosteric inhibitors that, however, would have to involve structurally distant sites for antibody and ligand binding.

### Inhibitory mAb475 and mAb926 recognize overlapping but distinct epitopes within the mannose-binding pocket of the active FimH conformation

**Fig 3:**
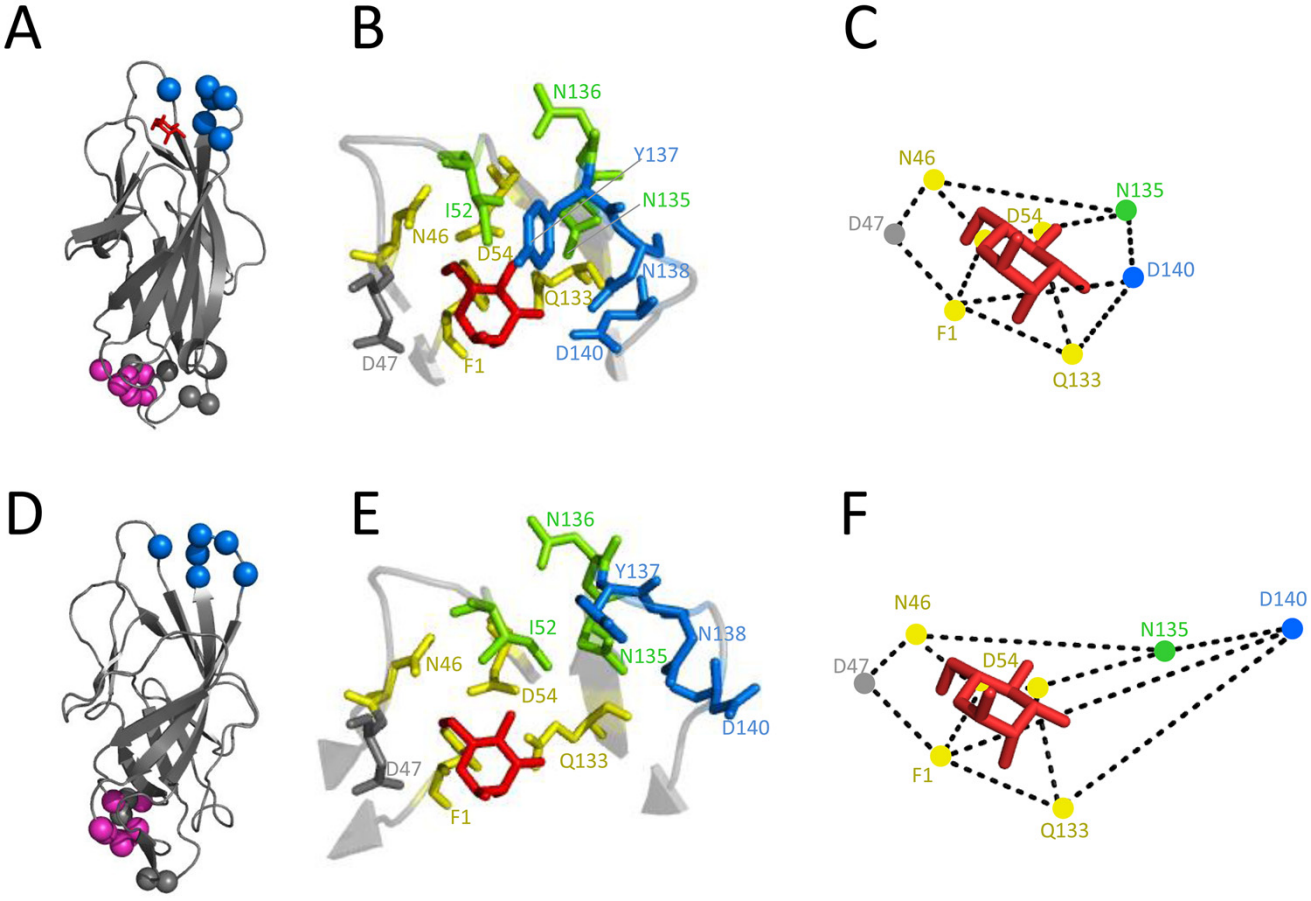
Inhibitory antibodies mAb926 and mAb475 recognize epitopes differently overlapping the mannose-binding pocket of FimH. Epitope of mAb926 mapped on crystal structure of the active (A) and inactive (D) conformations of the FimH lectin domain. Cα atoms of mAb926 epitope residues are shown as blue spheres. The natural allosteric site (residues V28, V30, S114 and A115)[11] is shown as grey spheres and D-mannose is shown as red sticks. The epitope of mAb21 is shown as magenta spheres. (B and E) Close-up of FimH binding pocket with mAb926 and mAb475 epitopes presented as colored sticks. The mAb926 epitope is shown in blue and mAb475 epitope is shown in yellow. The overlapping residues of these two epitopes are shown in green. D47 residue, involved in direct hydrogen bond with D-mannose but not being a part of either mAb epitope is shown as grey sticks. (C and F) Relative distances between mannose-contacting amino acid residues in the active and the inactive FimH conformations, respectively. The crystalized and the computationally docked (see text) positions of D-mannose (red sticks) are also shown. The presented distances were measured between heavy atoms of residue side chains that are known to form hydrogen bonds with D-mannose ligand [38]. The PDB codes for active- and the inactive structures are 1UWF and 3JWN, respectively. The position of D-mannose in the 1UWF was determined by alignment with the sugar ring of butyl α-D-mannoside of the original crystal structure.

We next determined the mAb926 binding epitope in FimH by site-directed mutagenesis (S2 Table) and compared it with the locations of the mAb475 epitope and mannose-interacting residues of the adhesin defined according to lectin domain crystalized in the high-affinity (active), mannose-bound conformation (Fig 3A).

Alteration of positions 52, 135–138 and 140 in FimH abrogated mAb926 binding (Table 1 and S2 Table). These positions form a compact epitope located on the top of the lectin domain (Fig 3A, blue spheres), i.e. on the side of the beta-barrel where the mannose-binding pocket is positioned. This epitope location is opposite from the domain-domain interaction interface that comprises the natural allosteric site of the lectin domain (Fig 3A, grey spheres) [11]. Three out of 6 of the residues in the mAb926 epitope, I52, N135 and N136, are also part of the mAb475 epitope (which include positions 1, 46, 52, 54, 133, 135, and 136) (Table 1 and Fig 3B) [33]. The predicted structural overlap of mAb926 and mAb475 epitopes is also supported experimentally by the fact that mAb926 and mAb475 strongly cross-interfere with each other’s binding to FimH (S4 Fig).

**Table 1:** The overlap between the FimH pocket residues and mAb926 and mAb475 epitopes.

FimH			α-man-FimH AA hydrogen bond distance (Å)^1^		
AA	Epitope		α-man atom	FimH conformation	
	mAb926	mAb475		Active	Inactive
Phe1		+	O2/O5 /O6	2.9/2.9/2.8	2.9/3/2.8
Asn46		+	O6	3.1	3
Asp47			O6	2.9	3.1
Ile52*	+	+	-	-	-
Asp54		+	O4/O6	2.5/2.5	2.7/2.6
Gln133		+	O3	3.1	3.1
Asn135	+	+	O3/O4	3.6/2.9	6.2/6.2
Asn136	+	+	-	-	-
Tyr137*	+		-	-	-
Asn138	+		-	-	-
Asp140	+		O3	2.6	9.9

Distances between mannose ligand and hydrogen bond forming FimH amino acids in the active and inactive conformation of the binding pocket are also shown.

^1^ Distance between hydrogen bond forming atoms of α-D-mannose and FimH amino acid residues in the active- (1UWF) and the inactive (3JWN) conformers of lectin domain as measured by PyMole.

*AA involved in hydrophobic interactions with mannose.

Epitopes of both antibodies overlap with the mannose-binding pocket (Table 1 and Fig 3B), in particular with the network of 7 side chain residues that form 11 hydrogen bonds with the ligand [38]. However, the mAb475 epitope is positioned on at least three different areas of the pocket (46–54 and 133–142 loops and N-terminal end) and includes a total of 5 of these mannose-interacting residues that form a total of 9 hydrogen bonds with the ligand [33,38]. In contrast, almost the entire mAb926 epitope is limited to just one side of the pocket formed by loop 133–142, with only two residues—135 and 140—forming a total of 3 hydrogen bonds with mannose. Consistent with structural data, mutation of residues that are directly (N135 and D140)- or indirectly (N138) involved in hydrogen bonds with ligand substantially decreased ligand binding [33,38,39], while mutation of remaining mAb926 epitope residues had no or only minor effect on the interactions with mannose [33].

Thus, the epitope of non-competitively inhibiting mAb926 overlaps with the mannose-binding pocket of the active FimH but, in contrast to the mAb475 epitope, is mostly limited to just one loop of the pocket. Still, because two residues of the mAb926 epitope contribute to the network of hydrogen bonds with the mannose ligand, it is plausible to expect some inhibitory effect of mAb926 against mannose binding, consistent with results reported above.

### The mAb926 epitope shifts away from mannose-interacting residues in the inactive FimH

While the overlap of mAb926 epitope with the mannose-binding residues in the active FimH explains the inhibitory potential of the antibody, it does not explain the non-competitive nature of the mAb926 inhibition. Thus, we turned to the alternative FimH structure (3JWN), where the lectin domain assumed a more twisted conformation and interacts with the pilin domain (Fig 3D). This structure was obtained in the absence of mannose ligand and its binding pocket is in an open, low-affinity (i.e. inactive) conformation.

As the inactive FimH structure was obtained in the absence of ligand, we first determined the potential position for mannose in the open configuration of binding pocket. Mannose was docked into the pocket of the 3JWN crystal structure using coordinates present in the active 1UWF structure followed by energy minimization using the CHARMM and the PARAM22 force field. As shown in Table 1 and Fig 3E mannose is predicted to take a position in the open pocket that retains 8 out of 11 hydrogen interactions of the active FimH with side chains of 5 out of 7 mannose-interacting residues—Phe1, Asn46, Asp47, Asp54 and Gln133 [38]. Interestingly, these five residues in the inactive binding pocket retain essentially the same position relative to each other as in the active FimH (Fig 3C and 3F), with the distance shift being 0.1 to 1 Å (0.44 Å ± 0.3 on average)(S5 Fig). This position of mannose is also supported by previous studies that employed molecular dynamics simulations of the active pocket or resolved crystal structure of mutationally inactivated FimH [38,40] (see Discussion).

According to the predicted position of mannose, two of the residues that interact with mannose in the active structure—Asn135 and Asp140—would lose their contacts with mannose in the open binding pocket (Fig 3E and Table 1). In the alternative FimH structures, these two residues also shifted significantly relative to one another and the other five mannose-interacting residues (Fig 3F), with the shift being 1.2 to 7.7 Å (3.9 ± 2.2 Å on average) (S5 Fig).

Thus, based on the projected mannose position in the inactive FimH, the mAb926 epitope residues that form hydrogen bonds with mannose in the active conformation are shifted relatively further away from the ligand in the inactive conformation. Thus, while in the active FimH pocket a portion of the mAb926 epitope is occupied by mannose, in the inactive FimH the entire mAb926 epitope is potentially accessible to the antibody.

### mAb926 blocks FimH conversion from inactive to active conformation

Because the mannose binding pocket of FimH is allosterically coupled with the rest of the lectin domain, we compared the effects of mAb926 and mAb475 antibodies on the conformation of FimH. For this, we used the mAb21 antibody that recognizes only the active conformation of FimH. The mAb21 epitope (Fig 3A, magenta spheres) is located distal to the mannose-binding pocket and close to the interdomain interface, presumed natural allosteric site (Fig 3A, grey spheres) [11].

**Fig 4:**
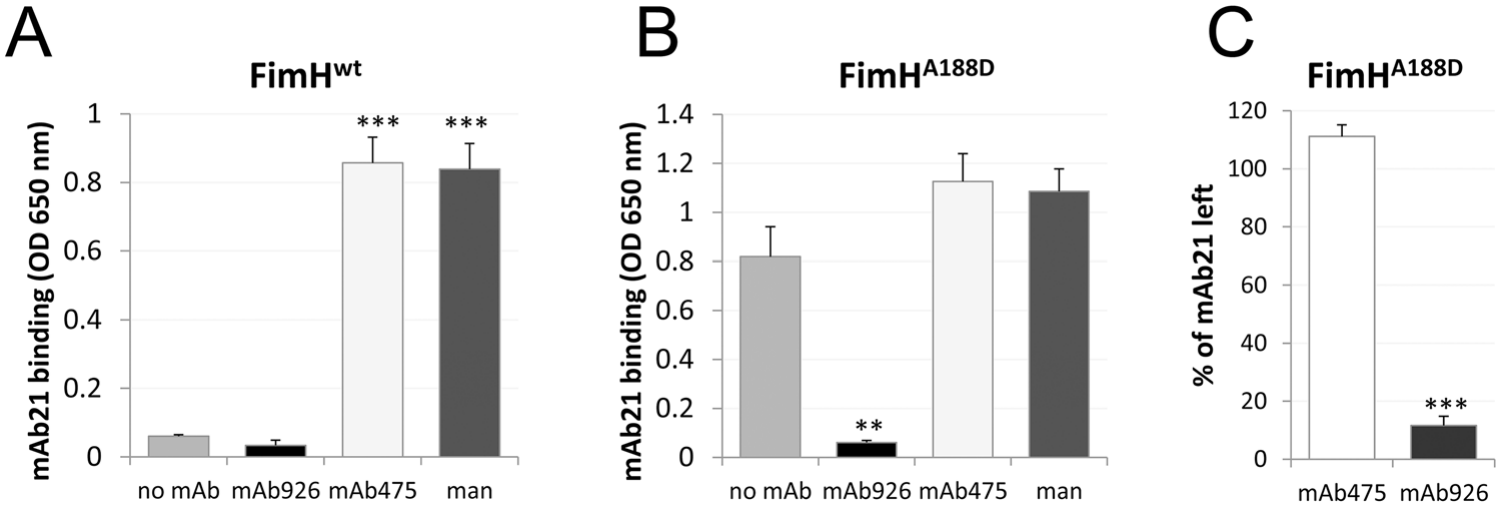
Effect of antibody binding on FimH conformation. Binding of the active state-specific mAb21 (biotinylated) to fimbrial FimH^wt^ (A) and FimH^A188D^ (B) after pre-treatment with 1% (52 mM) mannose, 50 μg/ml mAb475, or mAb926. (C) Elution of mAb21 (biotinylated) from mAb21-FimH^A188D^ complex 60 minutes after addition of mAb475 or mAb926. Data shown are mean ± SEM (n = 3 independent experiments). **, *P* ≤ 0.005, ***, *P* ≤ 0.0005 (t-test).

As shown in Fig 4A, binding of mAb475 and mannose to fimbrial FimH^wt^ converts it from the inactive to the active conformation as determined by binding of active conformation-specific mAb21. In contrast, mAb926 failed to induce such a conversion. We then performed the same experiment but using a FimH mutant variant that has the A188D mutation in the pilin domain that interferes with its interaction with the lectin domain and, in contrast to FimH^wt^, sustains FimH in active state even in the absence of mannose [13]. Pre-treatment of FimH^A188D^ fimbriae with mAb926 entirely abolished its recognition by mAb21 (Fig 4B), again in sharp contrast with the pretreatment with mAb475 which enhanced subsequent mAb21 binding. Moreover, when FimH^A188D^ was first pre-treated with mAb21 followed by incubation with the inhibitory mAbs, mAb475 stabilized mAb21 binding, while mAb926 resulted in almost complete displacement of the active state-specific antibody from the adhesin (Fig 4C).

Thus, not only is mAb926 binding to the FimH pocket unable to induce the shift from the inactive to the active conformation of the lectin domain, but it does the opposite—induces a shift away from the active conformation.

### Mannose can displace mAb926 but not mAb475 from FimH

We assessed whether soluble mannose and mAb926 could interact with the binding pocket simultaneously as predicted by the non-competitive inhibition model. We hypothesized that if mannose and mAb926 do bind together to FimH, then mannose should be able to bind to, and destabilize, a pre-formed complex of FimH with the antibody, resulting in a higher off-rate of mAb926. In contrast, this should not occur for mAb475 bound to FimH as the competitive antibody would fully prevent access of mannose to the pocket and the effect of mannose on the mAb475-FimH complex would be insignificant. In other words, the mannose effect after antibody binding will be opposite from the effect before/during the binding (Fig 2 above). Thus, we measured the effect of mannose ligand on the antibody-FimH complexes using surface plasmon resonance.

**Fig 5:**
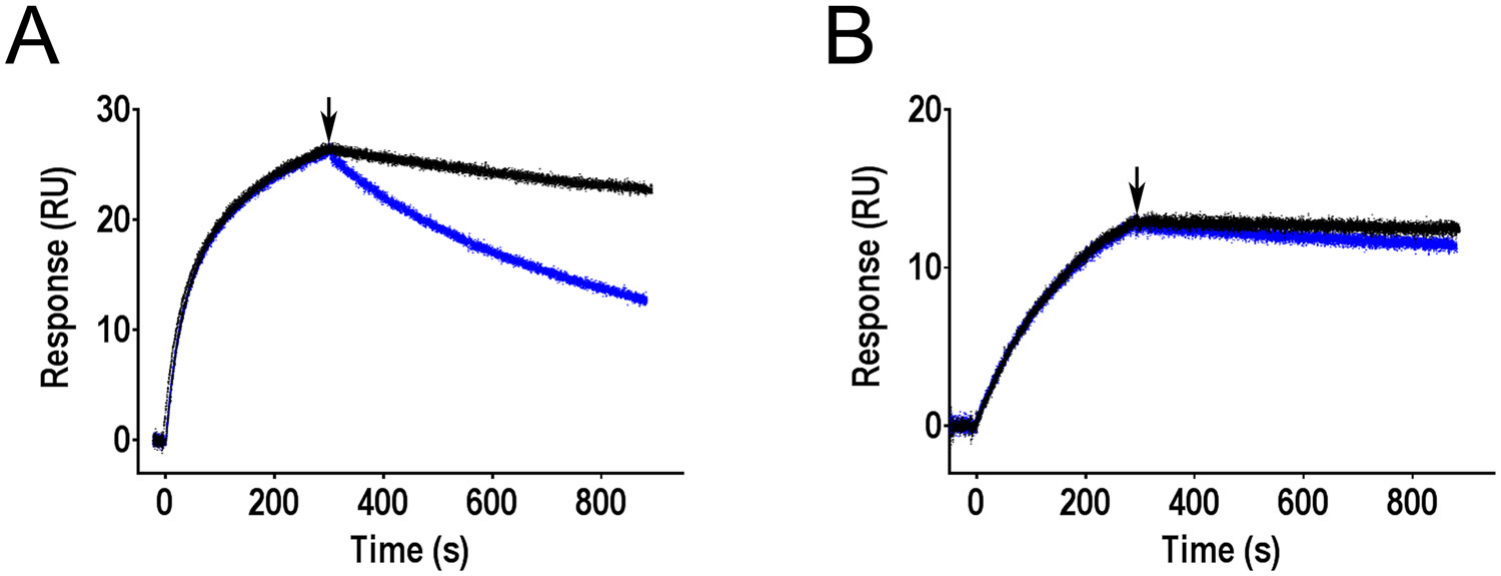
Effect of mannose on antibodies bound to FimH as determined by surface plasmon resonance. Binding of mAb926 at concentration 50 nM (A) and mAb475 at concentration 100 nM (B) to CM5 chip-immobilized fimbriae with FimH^wt^ was allowed to proceed for 300 s, and running buffer without or with 1% mannose (black and blue curves, respectively) were then injected to the flow cell at the time point designated by arrow, for 600 s. Duplicates for each antibody and conditions tested (+/- mannose) are shown.

Surface coated with FimH^wt^ fimbriae were first allowed to bind mAb926 or mAb475 and then antibody-FimH complexes were exposed to running buffer with and without a high concentration (1%) of soluble mannose. As shown in Fig 5, the dissociation rate of mAb926 from FimH was dramatically increased in the presence of mannose. At the same time, mannose had no significant effect on the stability of the complexes between mAb475 and FimH over the observed time period.

These results demonstrate that addition of soluble mannose affects the stability of the FimH-mAb926 complex and, thus, the antibody and the ligand must be able to bind to FimH simultaneously, consistent with the non-competitive nature of their interaction. In contrast, there is no such evidence for simultaneous interaction of mannose and mAb475 with FimH consistent with the direct orthosteric inhibitory mechanism of that antibody. Thus, the effect of mannose on the pre-formed antibody-FimH complexes was opposite from the antibodies effect on the complexes formation.

### mAb926 differs from mAb824 which allosterically stabilizes the low-affinity state of FimH

**Fig 6:**
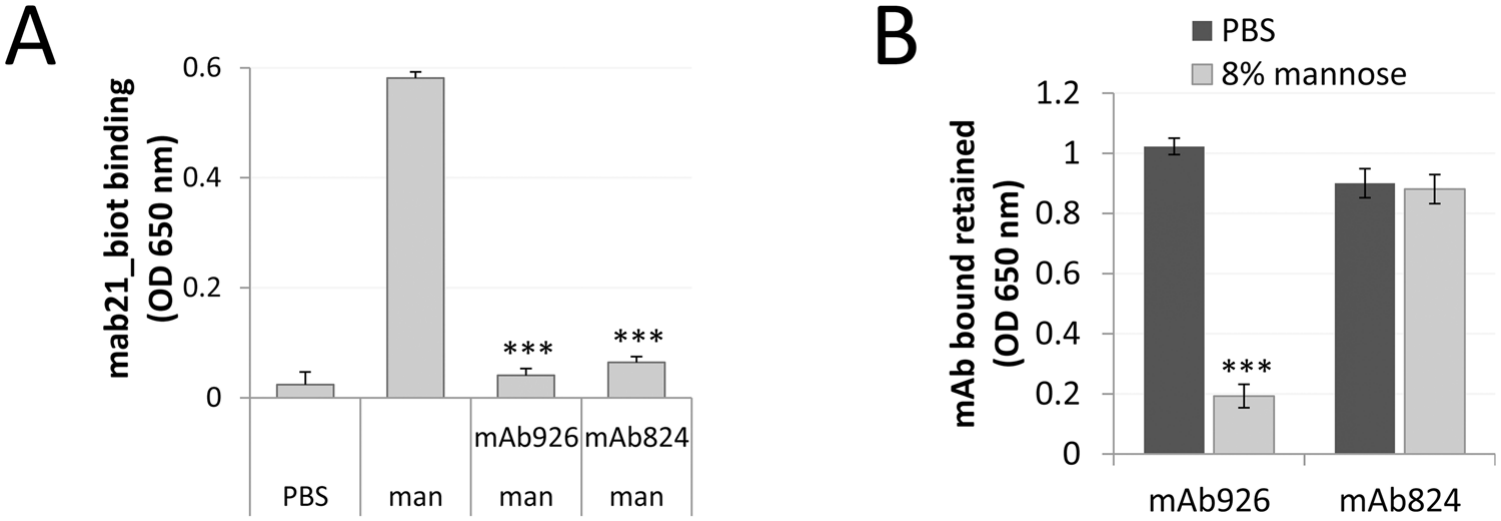
Comparison of the low affinity conformation-stabilizing mAbs. (A) Binding of the active state-specific mAb21 (biotinylated) in the presence of 0.01% (0.52 mM) mannose to untreated or mAb926- and mAb475-treated fimbrial FimH^wt^. (B) Level of antibodies bound to FimH^wt^ retained after treatment of the antibody-FimH complexes with PBS or 8% (440 mM) mannose for 1 h. Data shown are mean ± SEM (n = 3 independent experiments). ***, *P* ≤ 0.0005 (t-test).

Considering that mAb926 was found to stabilize the low-affinity conformation of FimH, we determined whether any antibodies from our original panel [33] have analogous activity. We found that indeed one of the antibodies, mAb824, can also prevent binding of active state-specific mAb21 to FimH^wt^ in the presence of soluble mannose (Fig 6A), i.e. mAb824 stabilizes the low-affinity state of the adhesin similar to mAb926. Unlike the latter, however, mAb824 recognized an epitope located away from the mannose-binding pocket (residues G79, S80, Y82, and P91; S3 Table) suggesting that the stabilization of the low-affinity conformation of FimH occurs via an allosteric, i.e. away-from-ligand, mechanism. While mannose could not displace mAb824 from FimH^wt^ in SPR experiments (S6A Fig), SPR studies with mAb824 could not be reliably performed due to difficulties in re-generating the antigen surface upon the mAb824 antibody binding. Thus, the stability of mAb926- and mAb824-FimH^wt^ complexes in the presence of soluble mannose was measured by ELISA. Unlike mAb926, mAb824 is not displaced from FimH^wt^ even at high (8%) concentration of ligand (Fig 6B). Notably, in the absence of mannose, both antibodies were binding to FimH at the same level upon 4 h-long incubation with PBS (S6B Fig).

These results suggested that while stabilization of the low-affinity conformation of FimH by antibodies could be achieved via an allosteric mechanism, the parasteric mechanism may provide unique properties of interference between the mannose ligand and mAb926 binding, not provided by an allosteric mechanism.

### mAb926 is superior to the competitive antibody, mAb475, in detaching surface biofilm and protection against urinary tract infection

**Fig 7:**
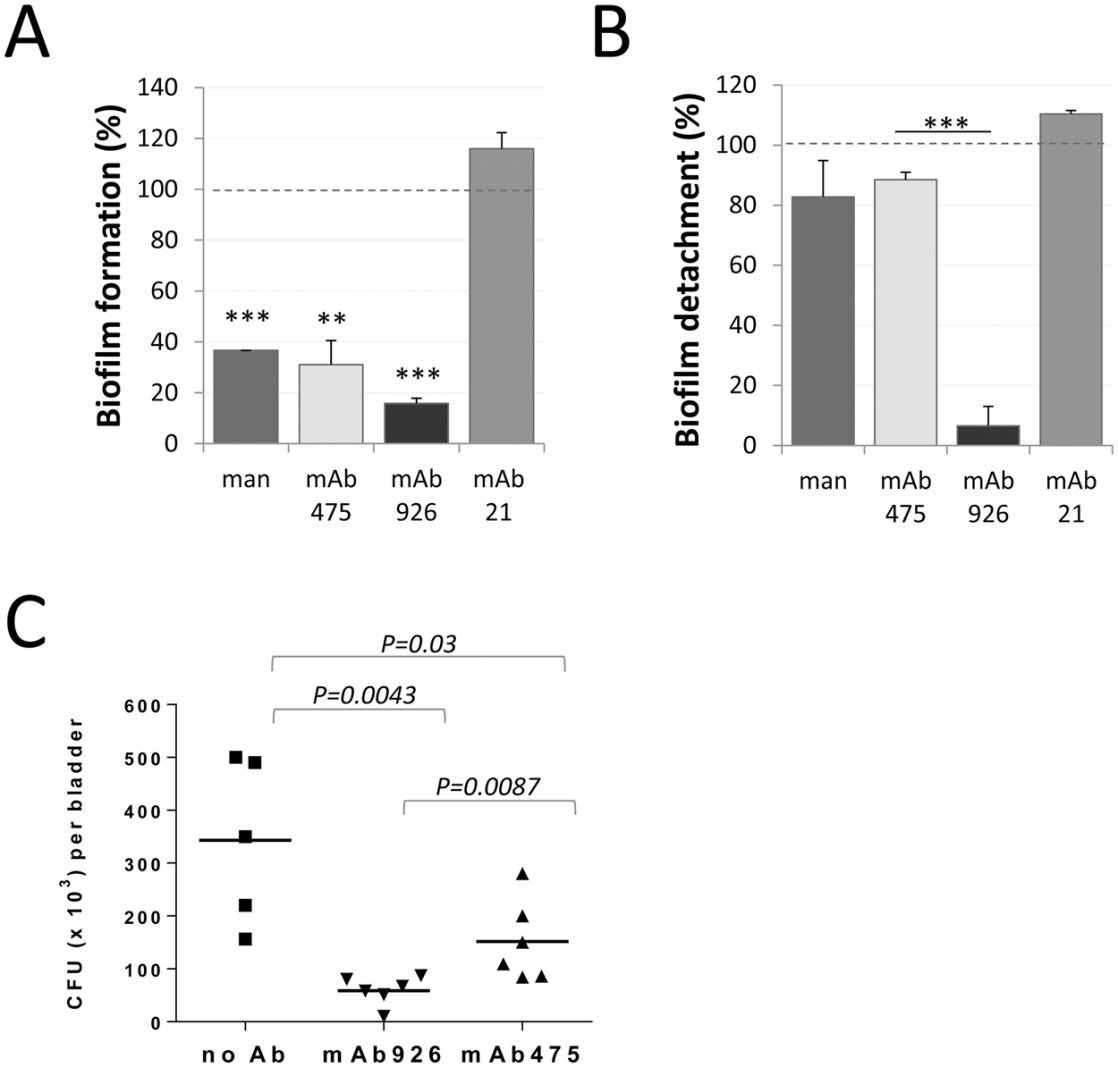
Effect of mAb926 and mAb475 on biofilm formed by *E*. *coli* UTI89 on mannan-coated surfaces and bladder infection in mice. (A) Biofilm formation by *E*. *coli* UTI89 in the presence of 1% mannose or 50 μg/ml monoclonal antibodies. Data (mean ± SEM, n = 4 independent experiments) are shown relative to the level of biofilm formed in the absence of mannose or antibodies (dashed line). **, *P* ≤ 0.005, ***, *P* ≤ 0.0005 (one sample t-test). (B) Detachment of 16 h-old *E*. *coli* UTI89 biofilm in the presence of 1% mannose or 50 μg/ml mAbs. Data (mean ± SEM, n = 3 independent experiments) are shown relative to the level of biofilm detached in the presence of PBS (dashed line). Biofilm was quantified using the crystal violet staining method. ***, *P* ≤ 0.0005 (t-test). (C) *E*. *coli* UTI89 recovered from bladders 24h post transurethral infection of mice. Bacteria, prior to administration into mouse bladders, were pre-incubated with PBS, mAb926 or mAb475 for 1 h. Horizontal bars indicate the mean (n = 5 or 6 mice per group). *P* values for indicated datasets were determined using two-tailed Mann-Whitney test.

We evaluated the effect of inhibitory antibodies against an *E*. *coli* biofilm formed on a mannose-coated surface by the model uropathogenic strain UTI89 expressing type 1 fimbriae. Both mAb475 and mAb926 (as well as soluble mannose) prevented formation of *E*. *coli* biofilm on a mannan-coated surface when they were added to the bacteria prior to growth over the surface (*P*<0.005), indicating that biofilm formation is dependent on mannose-specific bacterial adhesion (Fig 7A). However, when we first allowed the surface biofilms to form on a mannan-coated surface overnight and then added the antibodies (or mannose), neither mAb475 nor soluble mannose caused substantial detachment of the surface-attached biofilm (Fig 7B). In contrast, mAb926 resulted in effective dissolution of the biofilm (93% biofilm reduction vs 12% for mAb475, *P*<0.0005).

We then compared the two antibodies for their ability to block *E*. *coli* infection in vivo. As shown in Fig 7C, incubation of *E*. *coli* UTI89 with mAb926 prior to inoculation of mice via bladder catheter blocked bladder colonization more effectively than mAb475. There was an 83% reduction in bacteria recovered from bladder of mice infected with *E*. *coli* UTI89 bacteria that were pre-treated with mAb926 (Fig 7C), while inhibition with mAb475 was 52% (*P* = 0.0087).

Thus, the non-competitively-inhibiting mAb926 is more effective than is the competitively-inhibiting mAb475 in assays that are most physiologically relevant, such as detachment of biofilms and prevention of bladder infections by uropathogenic *E*. *coli*.

## Discussion

**Fig 8:**
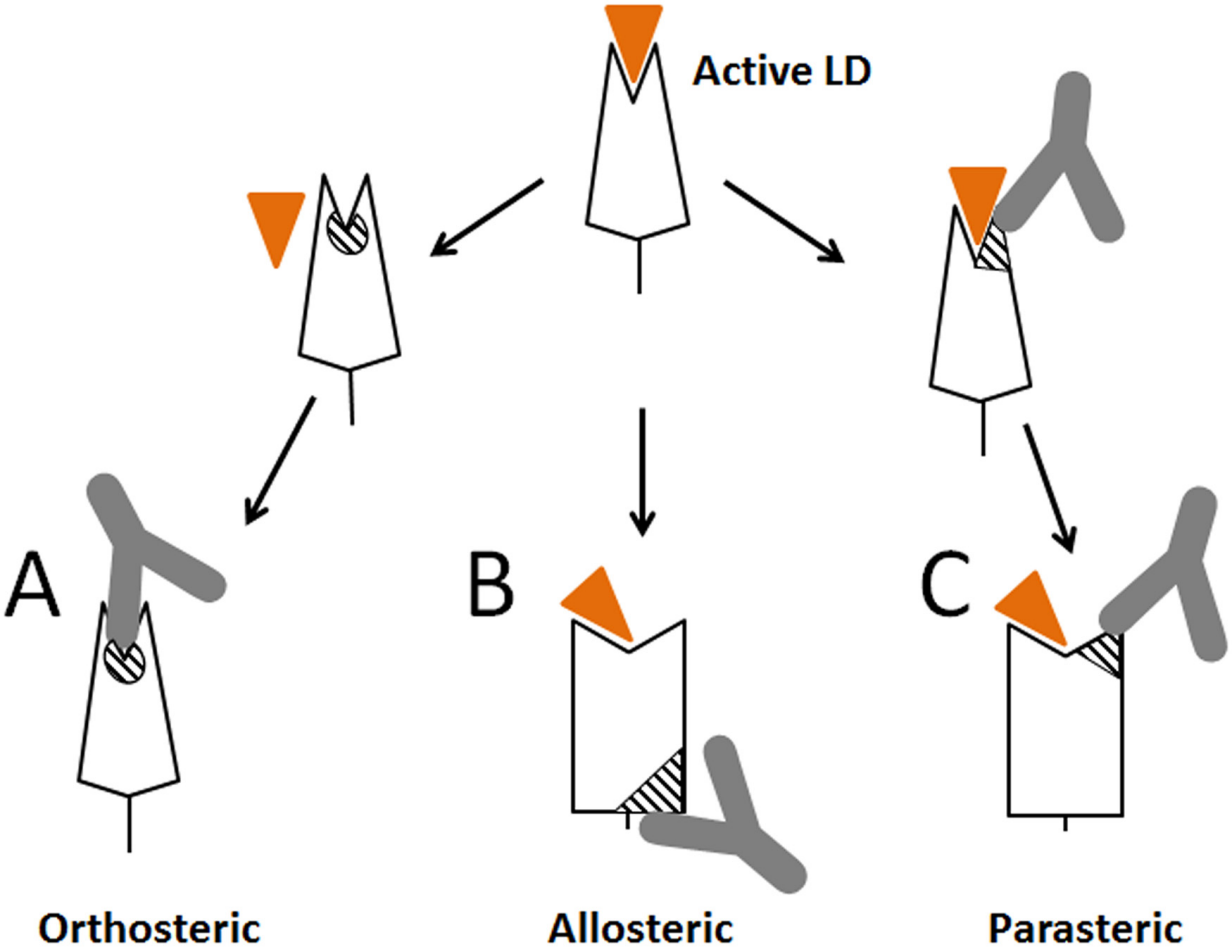
Schematic representation of different types of antibodies against lectin domain (LD) of FimH. (A) Orthosteric antibody. (B) Allosteric antibody. (C) Parasteric antibody. The triangular indent on the LD represents the mannose-binding site, and the orange triangle represents the mannose ligand. The striped elements represent the functional epitopes for orthosteric, allosteric and parasteric antibodies, respectively.

Creating antibodies targeting ligand-binding-site epitopes of receptor proteins is a primary goal in the development of protective or therapeutic antibodies. These antibodies are expected to block receptor binding functions by directly competing with the ligand. By definition, for competitive inhibition to occur, the binding pocket cannot be occupied by the ligand at the moment of inhibitor binding (Fig 8A). Thus, such orthosteric inhibitors cannot reverse ligand binding by triggering detachment of ligand from the pocket, and are ineffective in the presence of high concentrations of endogenous ligand, which limits their utility. The only inhibitors able to detach bound ligand are thought to be those of an allosteric nature that induce a weakly-binding inactive receptor state by signaling a conformational change from the site that is positioned distal to the ligand-binding pocket (Fig 8B). However, design of allosteric inhibitors is problematic for proteins where conformational regulation is unknown, not existing or complex.

We demonstrated here that the ligand-binding site of a receptor protein provides epitopes for powerful inhibitory antibodies that interfere with ligand binding within the pocket (like orthosteric inhibitors) but in a non-competitive manner (like allosteric inhibitors), via a mechanism that we refer to as parasteric (next-to-ligand) inhibition. Allosteric inhibitors have been described that bind near the pocket [41,42,43], however, unlike the parasteric inhibitor, they did not bind the ligand-interacting pocket residues themselves. The term ‘parasteric inhibition’ was suggested previously to highlight at least a theoretical possibility that inhibitor and ligand could bind in close proximity to each other rather than to fully overlapping or distant sites as expected for orthosteric and allosteric inhibitors, respectively [32]. That study was focused on modulation of enzymatic activity of ACP1 by purine modulators, but structural or mechanistic details of the inhibition were not examined. We show here that one of the striking apparent properties of the parasteric antibody is to bind to the binding pocket simultaneously with the ligand and prevent its conversion into the active state (Fig 8C). In this way, the parasteric concept is also distinct from the concept of inverse agonists, like those shown for the human G-protein coupled receptors, which can stabilize the inactive state of the pocket, but do not bind simultaneously with ligand [23,44].

Parasteric inhibition is potentially applicable to a wide range of receptors. Conformational dynamics of the binding pocket is considered to be an essential property of all ligand-binding proteins [45,46,47,48]. At least two different conformational states of the binding pocket are proposed to exist for receptor proteins—the active state that binds the ligand strongly and the inactive state that binds the ligand relatively weakly. In many or even the majority of cases, the active state pocket tightens around the ligand relative to the inactive state. For example, the two domains of the maltose binding protein hinge close to increase affinity [25,26], a ‘lid’ over the binding site of adenylate kinase closes to prevent substrate exit [49], a ‘gate’ of the plant hormone abscisic acid receptor closes around the hormone ligand [20], and the binding pocket of the beta-adrenergic receptor contracts around catecholamines [23,50]. Similarly, the ligand-binding loops of various receptor proteins have been shown to be highly flexible by NMR and FRET analysis [51,52]. This suggests that parasteric inhibitors could potentially bind simultaneously with ligand to the more loosely binding open pocket, preventing it from tightening in many different receptor-ligand systems. Notably, many of these receptors undergo only localized conformational dynamics that are not allosteric in nature, so the parasteric mechanism should be applicable to a wider range of receptors than the allosteric mechanism. The wealth of accumulated structural and functional data on different FimH states and the availability of various conformation-specific monoclonal antibodies provided an opportunity to use FimH as a prototype molecule to study dynamics of conformational shifts between active and inactive states and test various types of inhibitors and conformational modulators.

To gain insight into the mAb926 inhibitory mechanism, we turned to the crystal structure of inactive FimH with an open mannose-binding pocket, which was obtained without mannose [11]. Our previous studies on locking the inactive conformation have suggested that the open pocket of FimH retains some ability to interact with mannose [11]. Also, studies of others have shown that mutation of one of the mannose-binding residues, Gln133 to Asn, virtually eliminated the binding function of FimH, but mannose still could be co-crystallized with the mutant [38]. In the latter structure, mannose retained the same interactions as in the native active pocket with all binding residues except for the N135 and D140 residues that shifted away from the ligand, supporting our conclusion that the latter residues are on a flexible pocket loop. Further support for our model is provided by previous molecular dynamics simulations that predicted that residues F1 and D54 (i.e. those not on the flexible loop) form the strongest interaction with the ligand [40]. Thus, our model pointing to the dynamic nature of the 133–140 loop is consistent with previous studies.

It remains unclear whether the binding pocket must undergo a shift from the active to the inactive conformation in order for mAb926 (or any parasteric inhibitor in general) to gain access to its epitope when ligand is already bound. In the case of FimH, the 132–140 loop of the pocket that carries mAb926 epitope may transiently shift away from the attached mannose. Indeed, the intrinsic opening rate of maltose-binding protein has been recently shown to determine ligand dissociation [46]. Another possible scenario for mAb926 action could involve its intermediate binding to the active conformation of the mannose-occupied pocket of FimH via a portion of the epitope that does not involve the mannose-binding residues N135 and D140. In turn, the possible intermediate step of binding could facilitate the full opening of the flexible loop, thus de-activating the active state. Indeed, mutation of mAb926 epitope residues that are not involved in hydrogen bonding with ligand and facing outside the pocket cleft (I52, N136 and Y137) did not decrease or had only minimal effect on mannose ligand binding [33] suggesting that they could be accessible for interactions with the antibody even if the pocket is occupied by the ligand. Further studies are needed to elucidate the structural details of the inhibitory action of mAb926 and that of other potential parasteric inhibitors.

Although interaction of the lectin domain with the pilin domain in FimH allosterically facilitates the inactive conformation of the binding pocket, soluble pilin domain failed to stabilize the low affinity state of the adhesin [12]. In this study, however, we found an antibody, mAb824 that appears to prevent shifting of FimH^wt^ from the low- to high-affinity state in an apparently allosteric manner by binding an epitope located away from the binding pocket. This epitope is positioned on a side of the split sheet of the lectin domain of FimH that may be a critical region in the conformational pathway of the switch, but details of mAb824 action require further investigation. Importantly, we observed that, unlike mAb926, mAb824 is not displaced from FimH by soluble mannose suggesting that the ligand-induced conformational shift in FimH cannot overcome the conformation-stabilizing effect of mAb824. It is plausible to expect that at least in some receptor proteins, allosteric antibodies that are bound to the inactive conformation might be displaced by the ligand-induced activation of the protein and conversely that ligand bound to the active conformation might be displaced by allosteric inhibitory antibodies. However, allosteric antibodies are fundamentally different from parasteric antibodies in a way that is likely to affect the issue of induced dissociation. Allosteric antibodies bind to sites that are distinct from, and relatively weakly coupled to, the binding site [2]. This means that the antibody epitope can be in the inactive conformation while the binding site is in the active conformation. In contrast, a parasteric antibody by our definition has an epitope that to some extent overlaps with the binding site, so that binding kinetics must be changed if the antibody binds, and vice versa. We hypostatize that this difference explains the lack of mAb824 displacement by mannose from FimH but more extensive studies of mAb824 are needed to address this question. Thus, although mAb926 binding to FimH has an allosteric effect on the lectin domain, its inhibition of mannose-binding is not via the allosteric mechanism per se and is fundamentally distinct from the action exerted by mAb824 or other classic allosteric inhibitors described for other receptor-ligand interaction systems [42,43,53,54].

The model of non-competitive inhibition by mAb926 implies that the antibody and the ligand can bind simultaneously to FimH, i.e. form at least a transient FimH-mannose-mAb926 complex. This is experimentally supported by the observation that mannose accesses the FimH pocket occupied by mA926 but not by mAb475, resulting in unbinding of the former. The exact mechanism of how the three-way complex forms and why it is unstable is unclear, but is likely that antibody is eluted from FimH due to a structural distortion or steric hindrance caused by ligand binding. Reciprocally then, the co-binding property of mAb926 would lead to ligand unbinding when the receptor is already occupied by the ligand. Indeed, we observed that bacterial biofilm formed on a mannose-coated surface can be effectively detached only by mAb926 compared with the relatively minor effect of mAb475 antibody or even high concentrations of soluble mannose. To our knowledge, mAb926 is the only antibody shown to dissolve a bacterial biofilm.

The phenomenon of apparent simultaneous binding of the inhibitory mAb926 and the mannose ligand in spite of overlapping binding sites, makes it distinct from both the orthosteric and allosteric inhibitors, providing the rationale for defining a novel, parasteric mechanism of ligand binding inhibition. The advantage of parasteric inhibitors vs orthosteric inhibitors is that the former would be more potent in unbinding the ligand from the binding pocket and more effective in the presence of high concentrations of endogenous ligands. Either effect can explain the significantly stronger inhibition by mAb926 relative to the competitive inhibitor mAb475, and the superior ability of mAb926 to block FimH-mediated mouse bladder colonization. Although we demonstrated that the ligand can enter the binding pocket with bound mAb926 and in fact displace the antibody, this only occurred at very high, non-physiologic concentrations, and would not compromise the effectiveness of the antibody as a ligand-binding inhibitor. Many bacterial adhesins mediate shear-enhanced adhesion similar to FimH [55,56,57,58], suggesting that they may also undergo conformational changes and be candidates for parasteric inhibition. The advantage of parasteric inhibitors vs allosteric inhibitors is that the effectiveness of the parasteric inhibition is not limited by weak coupling of the allosteric site to the ligand binding site, and that parasteric inhibition does not require the receptor to be allosteric. In comparison to allosteric inhibitors which present difficulties for rational design due to lack of knowledge of the location of allosteric sites, development of parasteric inhibitiors may be more universally applicable to any protein with defined ligand binding sites, whether or not the protein is allosteric.

Taken together, our findings suggest that antibodies binding to just one loop of the ligand-binding site have the potential to be very effective for both inhibition and reversal of bacterial adhesion via the novel parasteric mechanism. The binding pocket side loops of bacterial adhesins appear to be good targets for generation of antibodies with such potential. The observation that the mAb926 epitope is largely formed by a single loop makes this loop a good candidate immunogen for induction of parasteric antibodies using synthetic cyclic peptides. Loop-shaped epitopes have been shown to be extremely potent as synthetic peptide—based vaccines due to their structural properties when synthesized in circular form that not only well mimic the native epitopes but also exhibit extremely high stability as immunogenic agents [59,60].

## Materials and Methods

### Ethics statement

All mouse work in this study was carried out in accordance with the recommendations in the Guide for the Care and Use of Laboratory Animals of the National Institutes of Health. The protocol no. 120304–03 was reviewed and approved by the New York University Institutional Animal Care and Use Committee.

### Bacterial strains

The *Escherichia coli* clinical isolate UTI89 and recombinant strains of *E*. *coli* K12 expressing type 1 fimbriae with different structural FimH variants were previously described [12,13,61]. Briefly, the recombinant strain of *E*. *coli* K12 (AAEC191A) carries pPKL114 plasmid containing the entire *fim* gene cluster from the *E*. *coli* strain K12, but with the inactivated *fimH* gene. For type 1 fimbriae expression, pPKL114 plasmid harboring bacteria were transformed with isogenic pGB2-24-based plasmids carrying different alleles of the *fimH* gene.

### Monoclonal antibodies

Hybridoma cell cultures producing mice anti-FimH monoclonal antibodies were described earlier [13,33]. Antibodies, mAb475, mAb926, mAb824 and mAb21 were purified from hybridoma culture supernatants using protein G-agarose (Millipore) according to manufacture recommendations. Germline origin of anti-FimH antibodies was determined based on the V-region sequence of their light chains [33] using IMGT/V-Quest software (http://www.imgt.org/IMGT_vquest/vquest?livret=0&Option=mouseIg) [34,35].

Mapping of mAb926 and mAb824 epitopes was performed as described previously [33]. Briefly, the antibodies were tested for the ability to recognize purified isogenic fimbriae carrying different mutations in LD of FimH. Parental (not mutated) fimbriae were used as a reference against which binding of the antibodies to all other mutant fimbriae was normalized. Epitopes of mAb926 and mAb824 were mapped using the high affinity FimH variant (FimH^wt:(186–201)FocH^, [12] and the low affinity FimH variant (FimH^wt^), respectively.

### ELISA assays

Microtiter plate wells were coated with purified fimbriae [33] at concentration 0.1 mg/ml in 0.02 M NaHCO_3_ buffer for 1 h at 37°C. The wells were washed with PBS and quenched for 20 min with 0.2% BSA in PBS. To test the effect of mannose on monoclonal antibody binding, immobilized fimbriae were incubated with serial dilutions of pure mAbs in the absence or presence of 52 mM α-methyl-D-mannopyranoside (αmm, hereinafter also termed ‘mannose’). Bound antibodies were detected with a 1:5,000 diluted HRP-conjugated goat anti-mouse antibody (Bio-Rad). The reaction was developed using 3,3′,5,5′-tetramethylbenzidine (TMB, KPL), and absorbance was read at 650 nm. EC_50_ values were determined by non-linear regression curve fitting using Prism 6.0 software (GraphPad, La Jolla, CA) for each antibody independently.

To test the effect of antibodies on the adhesin conformation, immobilized fimbriae were incubated with 50 μg/ml pure antibodies, or 52 mM mannose for 1 h and then 0.5 μg/ml biotinylated mAb21 was added to wells. After washing, binding of biotinylated mAbs was detected using a 1:5,000 diluted HRP-conjugated streptavidin (Sigma-Aldrich). In some experiments, surface-immobilized fimbriae were first incubated with 0.5 μg/ml biotinylated mAbs (in the absence or presence of 52 mM mannose) followed by incubation with purified mAbs 50 μg/ml for 1 h.

To test the effect of ligand on the stability of FimH-antibody complexes, antibodies at concentration 0.4 μg/ml were first bound to surface-immobilized fimbriae, followed by incubation with 8% mannose, or PBS for 1–4 h time.

### Surface Plasmon Resonance (SPR) analyses

SPR analyses of mAb926, mAb475 and mAb824 binding to FimH^wt^ followed by the absence or presence of 1% (w/v) mannose were conducted at 25°C in a running buffer (RB) of HBS-EP+ (0.01 M Hepes pH 7.4, 0.15 M NaCl, 3 mM EDTA, 0.05% (v/v) Surfactant P20) with 0.1 mg/mL BSA on a Biacore T100 system (GE Healthcare). Using standard amine coupling chemistry, ~2000 RUs of FimH^wt^ fimbriae were amine-coupled at 20 μg/mL in 10 mM glycine, pH 2.5 to 2 flow cells of a Series S CM5 chip (GE Healthcare). Two reference surfaces were prepared by activating and deactivating flow cells without the addition of protein. Duplicate (single for mAb824) samples at a single concentration were injected at a flow rate of 10 μL/min using a “dual” injection command in the T100 control software (v2.0.4) with injection 1 at 5 mins, injection 2 at 10 mins and a final dissociation time of 1 min. MAb alone curves were generated by injecting mAb followed by an injection of RB and double referenced [62] by subtracting a dual injection of RB followed by RB. mAb + mannose curves were generated by injecting mAb (in RB without mannose) followed by an injection of RB with 1% mannose and double referenced by subtracting a dual injection of RB followed by RB with 1% mannose. Optimal regeneration was achieved by injection of either one (for mAb926) or 2 (for mAb475) 30 second pulses of 10 mM glycine, pH 1.5 at a flow rate of 50 μL/min followed by a 2 min buffer stabilization phase. Optimal regeneration conditions for mAb824 sample binding were not found, and so required the generation of two FimH^wt^ fimbriae surfaces with only a single mAb824 injection on each. MAb926 and mAb475 injections with and without mannose were run on each FimH^wt^ fimbriae surface prior to the mAb824 injection in order to match mAb824 binding surfaces as closely as possible, as well as to provide a control for comparison. Sensorgrams were double-referenced in Scrubber 2.0b software (BioLogic Software), saved as text files, and re-plotted in Prism GraphPad 6 software.

To determine apparent kinetic rate and equilibrium binding constants, FimH^wt^ fimbriae were amine-coupled as noted above to a density of 1300 RUs, with an activated/deactivated surface used as reference. Serial 2-fold dilutions of analyte starting at 12.5 nM (mAb926) or 200 nM (mAb475), and buffer blanks were injected in random order and run in duplicate in HBS-EP+ with 0.1 mg/mL BSA at a flow rate of 30 μL/minute with 700 s of association and 1200 s of dissociation. Surfaces were regenerated with either one 30 s injection (mAb926) or two 30s injections (mAb475) of 10 mM glycine, pH 1.5 at 50 μL/minute followed by 2 mins of buffer stabilization. Double-referenced data were fit with a 1:1 binding model with BIAevaluation 2.0.4 software (GE Healthcare).

### Bacterial adhesion

Microtiter 96-well plates were coated with 20 μg/ml of yeast mannan (Sigma-Aldrich) in 0.02 M NaHCO_3_ buffer at pH 9.6. The wells were quenched with 0.2% bovine serum albumin (BSA, Sigma-Aldrich) in PBS for 20 min. Bacteria expressing FimH^wt^ (OD = 1) were first preincubated with different concentrations of mAbs for 1 h at 37°C and then allowed to adhere to mannan-coated surface for another 1 h. After an extensive washing with PBS, plates were dried and bound bacteria were stained with 0.1% crystal violet (Becton Dickinson) for 20 min at room temperature (RT). The wells were washed with water and 50% ethanol was added to the wells. The absorbance was measured at 600 nm.

### Biofilm formation assay

Microtiter 96-well plates were coated with 20 μg/mL of yeast mannan in 0.02 M NaHCO_3_ buffer at pH 9.6. Bacterial strains grown overnight in 3 ml LB media were spun and washed 1x with minimal essential media (MEM, Difco). Bacterial suspensions in MEM, at final concentration OD = 0.2, were added to mannan-coated wells in the absence and presence of 52 mM mannose or 50 μg/ml mAbs and incubated 16 h at RT without shaking. After washing with PBS, formed biofilms were stained with 0.1% (v/v) crystal violet (Becton Dickinson) as described above for bacterial adhesion assay. For biofilm detachment, 16 h-old biofilm produced by *E*. *coli* UTI89 on mannan-coated microtiter plates was washed 3 times with PBS and incubated in the absence or presence of 52 mM mannose or 50 μg/ml mAbs at RT with mild shaking. The wells were washed 3 times with PBS and stain for biofilm detection as described above.

### Mouse experiments

Infection of 10- to 11-week-old C57BL/6 female mice was performed as described elsewhere [33]. Briefly, bacteria were grown in LB medium without shaking for 48 h, harvested, washed twice in PBS and resuspended in PBS at a final concentration of 10^8^ CFU per ml. Bacteria were pretreated with 500 μg/ml mAbs for 1 h at 37°C prior to inoculation. Mice were anesthetized with ketamine/xylazine and twenty-five microliters of mAb-pretreated bacteria in PBS were inoculated transurethrally into mouse bladders via catheter. After 24 h, mice were sacrificed and bladders were aseptically removed and homogenized in 1 mL PBS. Serial dilutions were plated and total bacterial load per bladder was calculated. Statistical significance was determined using two-tailed Mann-Whitney test (GraphPad Prism 6.0 software, La Jolla, Ca).

### Modeling and visualization of protein structure

To dock α-D-mannose to the binding site of the inactive conformation of FimH, the crystal structure of the lectin domain (residues 1–158, with PDB code 3JWN) was aligned onto the high affinity structure of FimH lectin domain (PDB code 1UWF) followed by minimization of the RMSD of residues 1 to 6 and 44 to 48. The coordinates of mannose which are present in 1UWF structure were then used to create a complex between LD in low affinity conformation and the ligand. The entire system was then subjected to 100 steps of steepest descent minimization in vacuo and 500 steps of conjugate gradient minimization in a dielectric continuum using the program CHARMM.13 [63] and PARAM22 force field [64]. Based on published structural data, [38,65,66], the mannose-ring retains the same position and makes the same network of hydrogen bonds in the pocket, regardless the nature of mannosylated ligand (i.e α-D mannose, alkyl-derivatives of the mannose or oligomannose substrate). Thus, for simplicity, only the mannose-ring of α-D mannose was modeled and is presented in FimH structures.

In the 1UWF structure, α-D mannose was modeled in by alignment with the sugar ring of the mannose residue of the original crystal structure. The spatial distribution of amino acid residues involved in mAb epitopes and distances between atoms forming hydrogen bonds and mannose ligand in 3JWN and 1UWF structures were measured using the molecular visualization software program PyMOL (DeLano Scientific LLC).

### Statistics and data analysis

All values, unless otherwise indicated, are expressed as mean and SEM. Statistical significance was determined by two-tailed student test using Prism 6.0 software (GraphPad, La Jolla, Ca).

The receptor occupancy by antibody in the presence of mannose was calculated from the equation: [37]

$$EC_{50}^{ratio}=\frac{1+\frac{\left[M\right]}{K_{D}}}{1+\alpha \frac{\left[M\right]}{K_{D}}}$$

where, *α* is a cooperativity factor for mannose and antibody, [M] is mannose concentration and *K*_D_ is its equilibrium constant. For the strongest possible negative cooperativity (if mannose and antibody are direct competitors) *α* = 0 and ${\text{EC}}_{50\;}^{\text{ratio}}=1+\frac{\left[\text{M}\right]}{K_{\text{D}}}$.

As the dissociation constant for mannose and fimbrial FimH^wt^ is 298 ± 50 μM [13], and antibody binding was tested at a 52 mM concentration of mannose, the expected EC_50_ ratio for competitive binding is 175 ± 30.

## Supporting Information

S1 Fig Alignment of V-region amino acid sequences of mAb475 and mAb926 light chains. mAb475 is encoded by IGKV6-15*01 F and IGKJ2*01 F, and mA926 by IGKV1-117*01 F and IGKJ4*01 F alleles, respectively. Positions of the complementarity determining regions (CDRs, green boxes), and the clonal origin of the mAbs as determined by IMGT/V-Quest software.
(TIF)

S2 Fig Surface plasmon resonance measurements of antibody binding to CM5 chip-immobilized fimbriae with FimH^wt^. (A) Binding of mAb926. (B) Binding of mAb475. The experimental data (black curves) were fitted to a 1:1 binding model (red curves) using BIAevaluation 2.0.4 software (GE Healthcare). Duplicates of each concentration are shown.
(TIF)

S3 Fig Model of competitive (green field) vs non-competitive (blue field) binding of mannose and antibody to FimH receptor. R denotes FimH receptor, A denotes antibody and M denotes mannose. *K*_D_ and *K*_D(A)_ are respective equilibrium dissociation constants and α denotes the cooperative factor.
(TIF)

S4 Fig Inhibitory mAbs interfere with each other’s binding to FimH. Binding of biotinylated mAb475 (A) and biotinylated mAb926 (B) to the high affinity variant of FimH (FimH^wt:(186–201)FocH^, [12]) pre-incubated with PBS or designated antibody. The data shown are mean ± SD of triplicates from one representative experiment of multiple experiments that were performed with similar settings.
(TIF)

S5 Fig Distances between ligand-contacting residues in the active and the inactive conformations of the FimH binding-pocket. All distances are shown in Å and were measured between the heavy atoms of designated residues in the active- (PDB 1UWF) and the inactive- (PDB 3JWN) conformers of FimH by PyMol.
(TIF)

S6 Fig Binding to and dissociation of mAbs from fimbrial FimH. (A) Binding of mAb824 to CM5 chip-immobilized fimbriae with FimH^wt^ recorded by SPR. The mAb824 at concentration 200 nM was allowed to bind in two parallel channels for 300 s. At the time designated by the arrow, either running buffer (black curve) or running buffer with 1% mannose (blue curve) was injected for the next 600 s. Single replicate for each condition (+/- mannose) is shown. (B) Dissociation of FimH^wt^-bound antibodies upon 1–4 h-long incubation in PBS as determined by ELISA. Data are mean ± SD (n = 2 independent experiments).
(TIF)

S1 Table The binding parameters of mAb926 and mAb475 as measured by surface plasmon resonance. (RTF)

S2 Table Mapping of mAb926 epitope using FimH mutant library. (RTF)

S3 Table Mapping of mAb824 epitope using FimH mutant library. (RTF)
