# Supplementary figures and images for: Inhibition and Reversal of Microbial Attachment by an Antibody with Parasteric Activity against the FimH Adhesin of Uropathogenic E. coli
Source: PLoS Pathog. 2015 May 14;11(5):e1004857. doi: 10.1371/journal.ppat.1004857 (PMC4431754; doi:10.1371/journal.ppat.1004857)

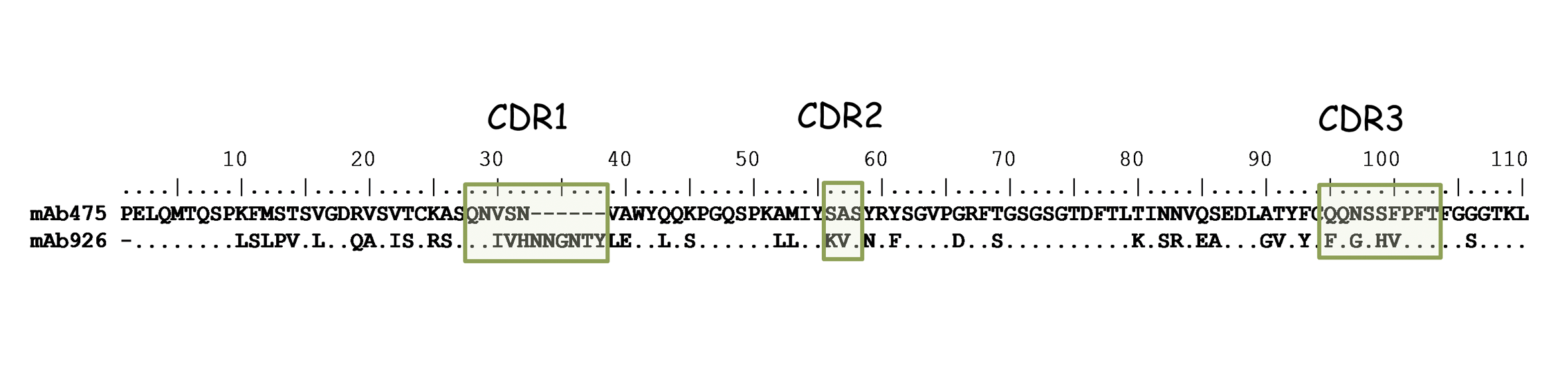

Supplement: S1 Fig — mAb475 is encoded by IGKV6-15*01 F and IGKJ2*01 F, and mA926 by IGKV1-117*01 F and IGKJ4*01 F alleles, respectively. Positions of the complementarity determining regions (CDRs, green boxes), and the clonal origin of the mAbs as determined by IMGT/V-Quest software. (TIF) [file ppat.1004857.s001.tif]

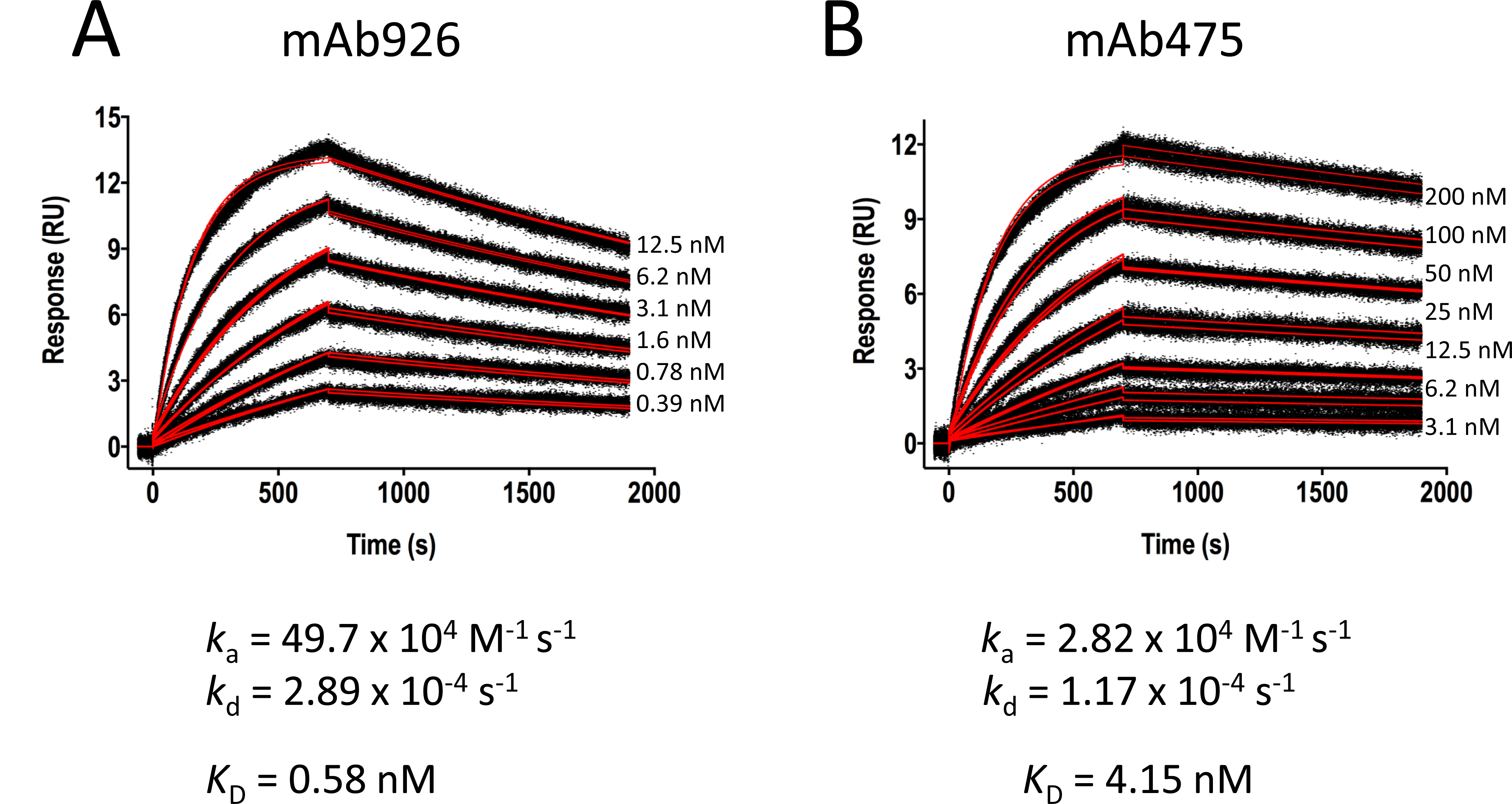

Supplement: S2 Fig — (A) Binding of mAb926. (B) Binding of mAb475. The experimental data (black curves) were fitted to a 1:1 binding model (red curves) using BIAevaluation 2.0.4 software (GE Healthcare). Duplicates of each concentration are shown. (TIF) [file ppat.1004857.s002.tif]

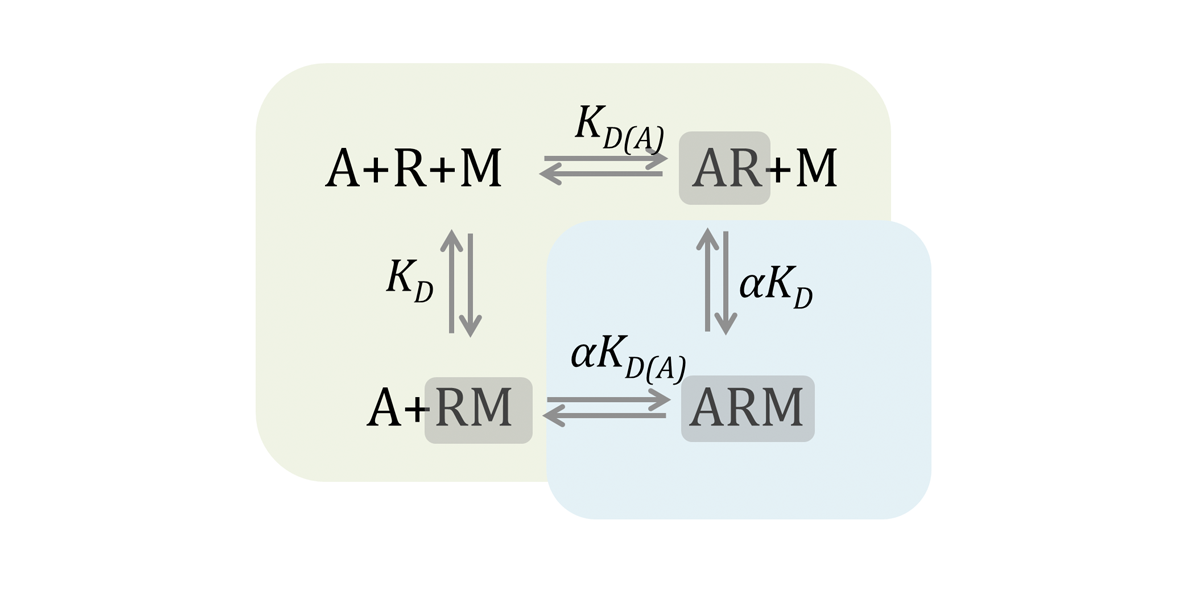

Supplement: S3 Fig — R denotes FimH receptor, A denotes antibody and M denotes mannose. K D and K D(A) are respective equilibrium dissociation constants and α denotes the cooperative factor. (TIF) [file ppat.1004857.s003.tif]

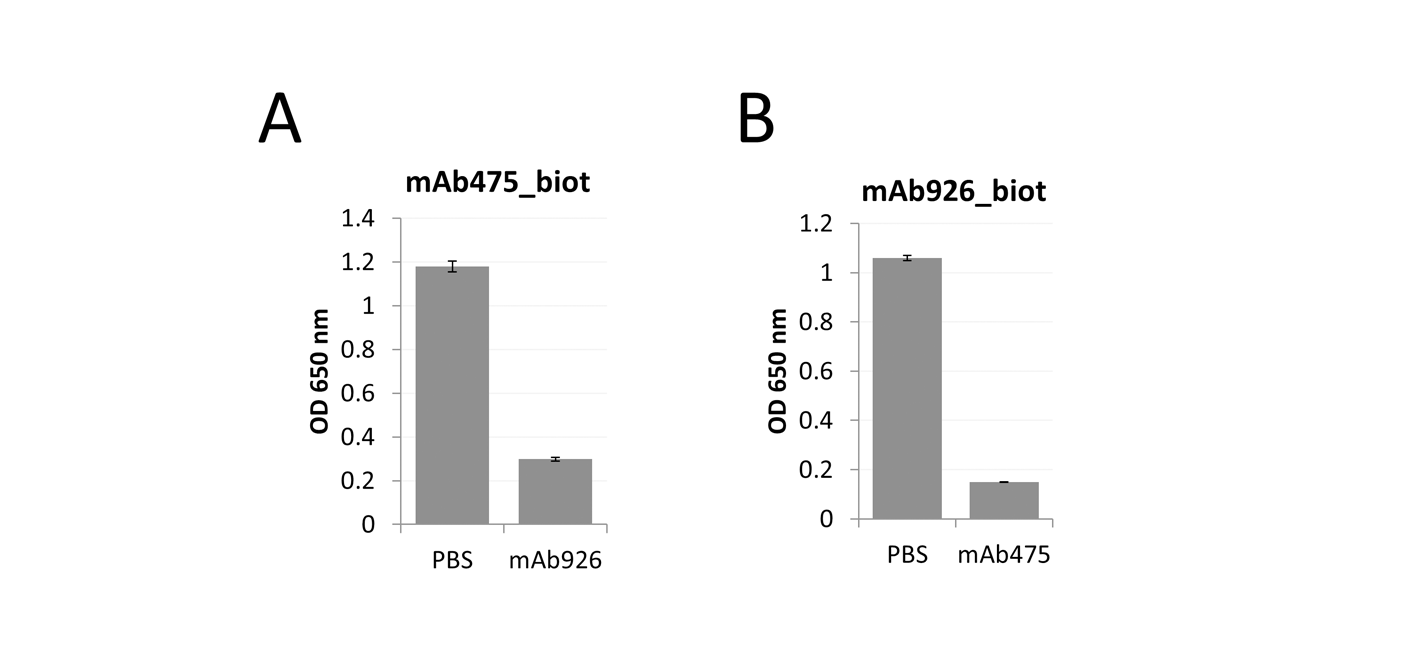

Supplement: S4 Fig — Binding of biotinylated mAb475 (A) and biotinylated mAb926 (B) to the high affinity variant of FimH (FimHwt:(186–201)FocH, [12]) pre-incubated with PBS or designated antibody. The data shown are mean ± SD of triplicates from one representative experiment of multiple experiments that were performed with similar settings. (TIF) [file ppat.1004857.s004.tif]

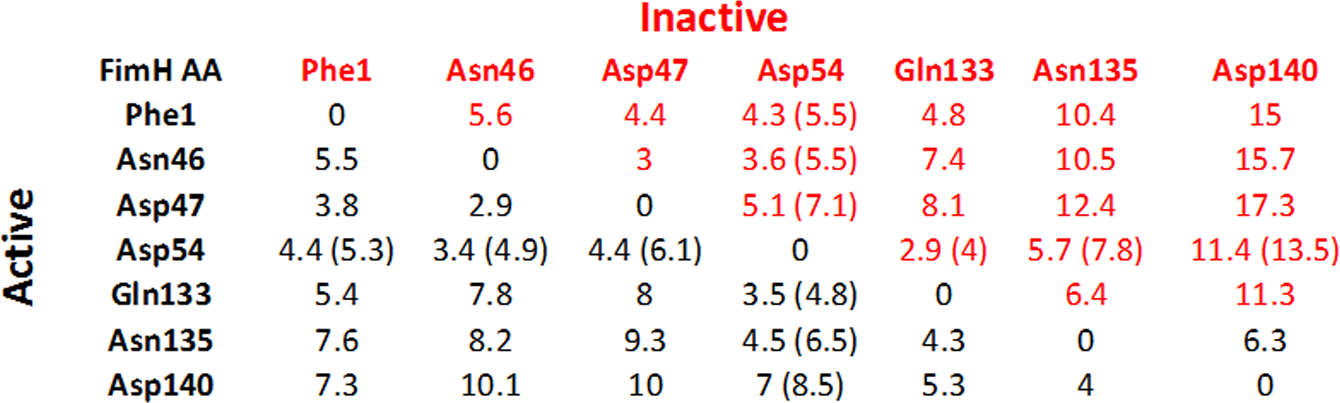

Supplement: S5 Fig — All distances are shown in Å and were measured between the heavy atoms of designated residues in the active- (PDB 1UWF) and the inactive- (PDB 3JWN) conformers of FimH by PyMol. (TIF) [file ppat.1004857.s005.tif]

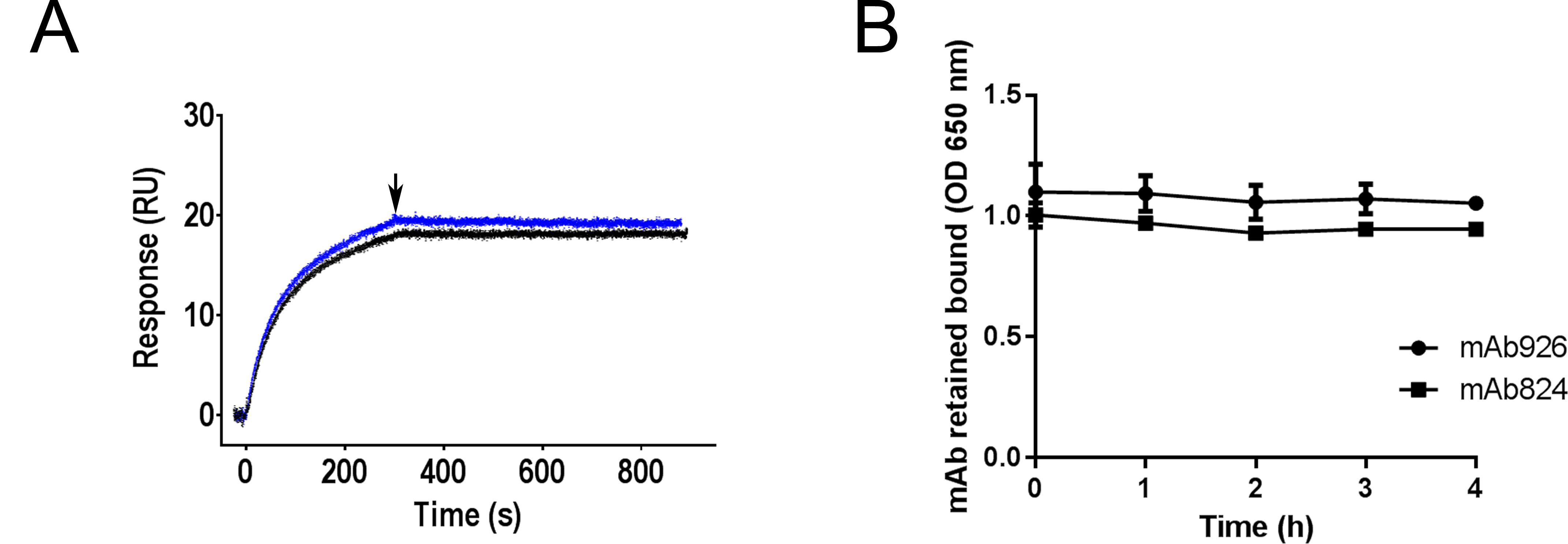

Supplement: S6 Fig — (A) Binding of mAb824 to CM5 chip-immobilized fimbriae with FimHwt recorded by SPR. The mAb824 at concentration 200 nM was allowed to bind in two parallel channels for 300 s. At the time designated by the arrow, either running buffer (black curve) or running buffer with 1% mannose (blue curve) was injected for the next 600 s. Single replicate for each condition (+/- mannose) is shown. (B) Dissociation of FimHwt-bound antibodies upon 1–4 h-long incubation in PBS as determined by ELISA. Data are mean ± SD (n = 2 independent experiments). (TIF) [file ppat.1004857.s006.tif]
