# Supplementary material for: Inhibition and Reversal of Microbial Attachment by an Antibody with Parasteric Activity against the FimH Adhesin of Uropathogenic E. coli
Source: PLoS Pathog. 2015 May 14;11(5):e1004857. doi: 10.1371/journal.ppat.1004857 (PMC4431754; doi:10.1371/journal.ppat.1004857)
Supplement: S1 Table — (RTF) [file ppat.1004857.s007.rtf]

Table S1. The binding parameters of mAb926 and mAb475 as measured by surface plasmon resonance.
	SPR1 
(data from Figure  S2)	SPR2*
	
	KD 
(nM)	ka 
x104 (M-1s-1)	kd 
x10-4 (s-1)	KD 
(nM)	ka 
x104 (M-1s-1)	kd 
x10-4 (s-1)	
mAb926	0.58	49.7	2.89	0.22	99.6	2.17	
mAb475	4.15	2.82	1.17	1.5	8.68	1.3	
mab926 vs mab475 (fold difference)	7.2 -
lower	17.6 -
highier 	2.5 -
highier	6.8 -
lower 	11.5 -
highier	1.7 -
highier	

*SPR2 measurements performed at the Analytical Biopharmacy Core, UW Seattle. Experiments were run in HBS-EP buffer using Biacore T100 system (GE/Healthcare). FimHwt fimbriae in 10 mM glycine, pH=2.6 were immobilized on a Series S CM5 Chip (GE Healthcare) at 1231 RUs using a contact time of 14 minutes. Following immobilization, a flow rate of 30 L/min, mAb analyte concentrations ranging from 0 M to 938 M were tested in triplicates using a contact time of 60 seconds and a dissociation time of 900 seconds.  Each run was followed with three 30 -second injections of glycine pH=1.5 to regenerate the surface.  Both the reference surface signal and the blank injection signal were subtracted from the resulting data, and the kinetic data “double referenced” in this manner was fitted globally across all concentrations to a 1:1 Langmuir binding model using the Biacore T200 Evaluation Software (GE Healthcare version 2.0).
