# Supplementary material for: Inhibition and Reversal of Microbial Attachment by an Antibody with Parasteric Activity against the FimH Adhesin of Uropathogenic E. coli
Source: PLoS Pathog. 2015 May 14;11(5):e1004857. doi: 10.1371/journal.ppat.1004857 (PMC4431754; doi:10.1371/journal.ppat.1004857)
Supplement: S2 Table — (RTF) [file ppat.1004857.s008.rtf]

Table S2. Mapping of mAb926 epitope using FimH mutant library. 
Mutation	mAb binding relative to wild type* (%)	
F1L	111	
A2S	92	
P12A	87	
I13S	66	
H45A	91	
N46A	90	
N46Q	104	
D47S	97	
Y48A	58	
E50A	69	
T51A	96	
I52A	19	
T53A	100	
D54E	119	
Y55A	99	
T57G	96	
I130A	82	
R132D	102	
Q133N	100	
T134G	122	
N135I	6	
N136A	13	
Y137A	2	
N138I	8	
S139A	105	
D140A	21	
D141A	73	
F142A	100	

* Binding of mAb926 to purified isogenic fimbriae with different mutations in LD of FimH (FimHwt:(186-201)FocH) was tested as described in Materials and Methods. Predicted mAb926 epitope residues mutation of which reduced the mAb binding >25% (and which also clustered together on FimH crystal structure) are marked in red. 
