# Supplementary material for: Inhibition and Reversal of Microbial Attachment by an Antibody with Parasteric Activity against the FimH Adhesin of Uropathogenic E. coli
Source: PLoS Pathog. 2015 May 14;11(5):e1004857. doi: 10.1371/journal.ppat.1004857 (PMC4431754; doi:10.1371/journal.ppat.1004857)
Supplement: S3 Table — (RTF) [file ppat.1004857.s009.rtf]

Table S3. Mapping of mAb824 epitope using FimH mutant library. 
Mutation	mAb  binding relative to wild type* (%)	
F1L	99	
A2S	102	
P12A	99	
I13S	98	
N46Q	97	
N46A	103	
D47S	105	
Y48A	104	
E50A	97	
I52A	98	
T51A	98	
D54E	100	
L68V	92	
S69C	65	
N70C	89	
N70G	93	
F71A	105	
S72A	99	
T74K	93	
G79R	4	
S80R	2	
S81R	95	
Y82A	3	
P83S	99	
P83R	98	
F84S	101	
P85S	99	
T87A	107	
T90G	100	
T90N	95	
P91A	98	
P91R	2	
R92A	108	
R92D 	99	
P104W	102	
I130A	105	
R132D	96	
Q133N	99	
N135I	109	
N136A	98	
Y137A	97	
I138A	86	
S139A	102	


* Binding of mAb824 to purified isogenic fimbriae with different mutations in LD of FimHwt was tested as described in Materials and Methods. Predicted mAb824 epitope residues mutation of which reduced the mAb binding >25% (and which also clustered together on FimH crystal structure) are marked in red.
